# Women in Dentistry

**Published:** 1877-03

**Authors:** 


### WOMEN IN DENTISTRY.

The following, though written several years ago, is so pertinent, and directly to the point, that we gladly publish it now. It is from the pen of one who has given much thought to the subject, and well understands whereof he writes.—[Ed.

Numerous inquires respecting ladies entering the dental profession have induced me to send you a few facts with reference to those now practicing, and other matters of interest connected therewith. If you think them worthy of a place in the Register, they may answer an inquiry in some mind, or suggest a profitable and satisfactory employment to some woman of energy. Of the various occupations now opening to women, there are none I think which promise a more certain compensation than does this profession, though few have yet chosen it owing to its novelty, and the various obstacles to be overcome in its acquirement, yet those who have met with a degree of success which once recognized, will certainly stimulate others to make a similar effort with an equal chance of success

In the autumn of 1864, Miss Lucy B. Hobbs, from Western New York, matriculated in the Ohio Dental College. She was the first lady admitted as a student in any dental school, and though surrounded by embarrassments from the novelty of her position, the following extracts from a letter received some time since from the Dean of that institution, shows with what courage and energy she conducted herself through a two years course of study, receiving a diploma in February, 1866. "A woman of great energy and perseverance, studious in her habits (missing but one lecture during the session and that at the request of the professor of anatomy), modest and unassuming in her manners. She had the respect and kind regards of every member of the class and faculty. * * * As an operator in the clinic room she was not surpassed by her associates, and often by them was both her opinion and assistance sought in difficult cases. * * * In the final examination she had no superior. * * * The class of which she was a member, though one of the largest ever in attendance, excelled all previous ones in good order and decorum, a condition largely due to the presence of the lady." Having received her diploma, she opened an office in Chicago, and there practiced successfully, until her removal to Lawrence, Kansas, where she now enjoys the reputation of having the most lucrative practice in the state."

In the fall of 1867, Mrs. Henrietta Herschfeld, a lady from Berlin, Prussia, presented herself as a student to the Pennsylvania College of Dental Surgery in Philadelphia; but it was only after much deliberation and many misgivings as to the result, on the part of a number of the professors, that she was admitted upon an equality with the male students. Nor did this objection arise so much from the objection to ladies studying dentistry, as from the fact of its being an innovation, and the unwillingness to meet the embarrassments that *might* arise from educating the sexes together. Before her college course had terminated, however, she had by her womanly bearing, and professional skill, won from the faculty and her classmates, a full recognition of her worth; and the united testimony of all was, that at no previous session had good order prevailed to such an extent, or the standard of clinical operations been higher.

The quite and gentlemanly bearing of the male members of the class during her presence in the school, was in such contrast with the boisterous and rowdy spirit for which the medical students of Philadelphia have been so notorious, that it was observed by all visiting the college.

Our German friend graduated March 1st, 1869, standing in the final examination unsurpassed.

Returning to her native country, and directing her attention especially to the teeth of children, the result of her experiment remained not long in doubt. Newspaper men, eager for an item, made her the subject of extended articles, thus bringing her into public notice, and though but a comparatively short period has elapsed since she opened her office, she now enjoys a patronage of which many practitioners of years standing might well be proud. At the risk of violating the sacredness of a private correspondence, I make the following extracts from a letter received some weeks since in answer to several inquiries respecting the motives which influenced her in the selection of a profession, and her course since in practice. To the first she says: "Frequent suffering in my own teeth, and while in the dental chair, without much relief, created the wish in my heart to understand the thing myself, and vanished when I had, very young to be the head of a very large country household. After being left a widow and thrown upon my own resources, I looked to see how women supported themselves. I saw how they were shut out from many occupations suited to their sex and abilities, and how they had to work for a trifle, or how ill they were treated because the few places open to them were over filled. Fully convinced that this state of affairs had to be changed in many respects, I concluded to do my part in taking up and carrying out my old favorite idea; so I came to Philadelphia with full confidence in the liberal minded Americans. * * * It was a trial for me to leave the good city where I had received so much kindness, but I felt my mission was at home.

"Thanks be to heaven! I have not been mistaken in my people, nor the wants of my time. It is now nine months since I opened my office, and my success has far exceeded my expectations. Nearly half of my patients belong to our aristocracy, and two of the royal princesses are among them. * * * The journals have spread my history over the continent, and from all parts of the country I receive letters of thanks and encouragement. * * * Mothers are delighted that I take especial care for children, and they place their little ones with confidence under my charge.

"I don't think many of my professional brethren like it much that the females have crept into their privileges, but I can't help the poor fellows, they will have to get used to it. * * * Besides my rich patients, I have to work for many poor but well educated persons, who can not afford to pay the prices of other American dentists. I am willing to do all I can for them, though such patients and their children take much of my time, without much pay; still I shall not think it difficult to make three or four thousand dollars a year in my office. I believe few women do so well, and then I have the evenings to myself for amusement and study.

"I am anxious to see the first female physician located here; the public mind is sufficiently prepared for it, and I am working for our cause daily."

Thus these ladies, with two others practicing in this country, having demonstrated their efficiency in this branch of the healing art, prove beyond a doubt, that the profession of dentistry, though almost monopolized by men, is not incompatible with the tasts and capacities of women.

There is probably no profession where the increasing demand for educated practitioners is so good as in this country; and any earnest, intelligent woman, with an ordinary amount of mechanical ingenuity, placing herself with some good dentist as a private pupil for a few months, and afterward passing through a reputable dental college, obtaining the degree of Doctor of Dental Surgery therefrom, is the possessor of a means of gaining a competency, and position in society afforded by few occupations.

The united testimony of the two institutions where ladies have been admitted, is so strongly in favor of their presence as a modifying influence upon the morality of the class, that they can not long be kept out of any dental college, and must ere long lead other institutions to objure the distinction of sex.

Of the dentists in this country, numbering near ten thousand, and consuming in their labor about three millions dollars of gold annually, with gross receipts exceeding twenty millions in currency, scarcely a fiftieth part of them possess an education requisite to a skillful practitioner, and this small minority receive as a reward for their skill more than two-thirds of this gross amount.

Dentistry, though a specialty, might be divided into operative, or surgical, and mechanical. The former embracing all of those operations intended for the improvement and preservation of the natural teeth, and must as dentists improve in education and professional skill, include oral surgery complete. All of which will require a series of delicate manipulations not inconsistent with refinement, and will furnish much of the most remunerative portion of the dentist's employment. The latter is confined to the insertion of artificial teeth, where the natural ones have been lost by accident or disease, and is of itself a business of importance and magnitude. One establishment alone, S. S. White's, of Philadelphia, makes four and a half millions of teeth annually, while there are many others, not so extensive in their capacity, though propably making half as many, increasing the number made in this country, (which supplies the world) at not much less than seven millions annually. These teeth are furnished the dentists by the dental depots throughout the land, and of these, a colored woman, Mrs. A. A. Fayerweather, formerly Mrs. Jennings, has one of the most successful, established about fifteen years since, by herself and husband, in New Orleans. From this point she supplies the dentists in that section of country with gold, teeth, instruments, etc., making an annual visit to Philadelphia, there to replenish her stock of materials from the manufactories in that city. It was the display of these articles for which she took the first premium at the state fair at Houston, Texas, some months since, and not for work as a dentist as the papers stated.

